# Early Changes in Visual Quality and Corneal Structure after DMEK: Does DMEK Approach Optical Quality of a Healthy Cornea?

**DOI:** 10.1155/2018/2012560

**Published:** 2018-09-23

**Authors:** Maria Satue, Miriam Idoipe, Alicia Gavin, Maria Romero-Sanz, Vasilios S. Liarakos, Antonio Mateo, Elena Garcia-Martin, Alejandro Blasco-Martinez, Antonio Sanchez-Perez

**Affiliations:** ^1^IIS-Aragon, Aragon Institute for Health Research (IIS Aragón), Zaragoza, Spain; ^2^Ophthalmology Department, Miguel Servet University Hospital, Zaragoza, Spain; ^3^Ophthalmology Department, Naval Hospital, Athens, Greece

## Abstract

**Purpose:**

To evaluate early changes in visual function and visual quality parameters after Descemet membrane endothelial keratoplasty (DMEK) and to compare the outcomes with healthy controls.

**Methods:**

Thirteen patients who underwent DMEK and 14 controls were evaluated. All subjects underwent visual function evaluation, including visual acuity under photopic and mesopic lighting conditions and contrast sensitivity (CSV) tests CSV 1000 and Pelli-Robson. Corneal parameters were assessed with Oculus Pentacam. Corneal mean keratometry (Km), corneal densitometry values, and low and high order aberrations (LOA and HOA) were recorded. In DMEK patients, all tests were performed before surgery and 1 and 6 months after surgery.

**Results:**

In patients who underwent DMEK, photopic visual acuity improved from 0.59 to 0.31 at 1 month (*p*=0.013) and 0.13 at 6 months (*p*=0.008); mesopic visual acuity and all contrast sensitivity values (both CSV and Pelli-Robson test) improved significantly in the first month (*p* < 0.005). A significant decrease was observed in corneal density in the 0–2 mm ring (from 43.83 to 35.60, *p*=0.043) and mean posterior Km (from −5.84 to −6.80, *p*=0.005) in the first month. Corneal HOAs and all corneal densities improved at 6 months after DMEK (*p* < 0.05). All visual function parameters and corneal aberrations remained lower and higher, respectively, compared with healthy controls (*p* < 0.05). Corneal densities were comparable with controls at 6 months after DMEK (*p* > 0.05).

**Conclusions:**

Patients undergoing DMEK present visual function improvement and a decrease in corneal density at 1 month after surgery. Decrease in corneal posterior HOAs can be observed at 6 months. However, visual function outcomes and corneal aberrations remained worse compared with healthy controls.

## 1. Introduction

In the last decade, selective replacement of the diseased endothelium with a donor endothelial graft has superseded traditional full-thickness penetrating keratoplasty [1], in the treatment of endothelial disorders such as Fuchs endothelial dystrophy and pseudophakic bullous keratopathy. Benefits of endothelial keratoplasty (EK) over penetrating keratoplasty include superior biomechanical integrity, faster visual recovery with better uncorrected visual acuity, and a more predictable refractive outcome with less induced astigmatism [2–4].

It has been well established that Descemet membrane endothelial keratoplasty (DMEK) produces better visual outcomes than other EK techniques. Theories explaining this improvement in visual results include a more regular posterior graft surface with greater thickness uniformity [5–7], thinner grafts with a better match in curvature, improved parallelism between the graft and recipient, and improved optical compensation by the posterior cornea [8]. These advantageous structural results might be explained by the transplantation of only an isolated Descemet membrane and its endothelium in DMEK, apparently resulting in near-normal anatomic corneal restoration. Thus, it has been suggested that a transplanted DMEK cornea may approach the optical quality of a healthy cornea [9].

Corneal aberrations after DMEK have been previously studied [9, 10]. However, research on visual function (which overall provide more accurate information on the patient's visual performance than high contrast visual acuity) and other corneal parameters such as corneal light scatter after DMEK is scarce, and surgery outcomes are mostly compared with other keratoplasty techniques rather than healthy controls. The purpose of the present study was to provide further and complete information on early changes in visual function and corneal parameters after DMEK and to compare visual and structural outcomes with healthy controls. This is the first longitudinal follow-up study in DMEK patients including all these visual function tests and densitometry analysis.

## 2. Methods

Thirteen patients who underwent DMEK surgery and 14 healthy controls were included in the study. All procedures adhered to the tenets of the Declaration of Helsinki, and all participants provided informed consent to participate in the study. The protocol and informed consent were approved by the local ethics committee for scientific research in Aragón (Comité Ético de Investigaciones Científicas de Aragón-CEICA, PI16/0010).

Pseudophakic patients with different stages of corneal edema secondary to endothelial disease (FED/BK) were selected for the study. The reason to include only pseudophakic patients was to avoid any alterations in the visual quality measurements caused by lens opacifications (cataract of any kind). DMEK surgery was programmed in all cases. The Descemet endothelial grafts were harvested from 13 donor corneoscleral buttons using the standardized “no touch” technique for endothelial graft preparation [11]. All DMEK procedures were carried out following the standardized “no touch” technique. To summarize, a descemetorhexis was performed up to 1 mm from the limbus under air. An anterior chamber maintainer (Centurion Vision System, Alcon laboratories Inc.) with continuous air infusion was used to fill the anterior chamber with air during descemetorhexis. The donor Descemet endothelial roll was inserted into the anterior chamber of the patient with a glass injector after staining with 0.06% trypan blue. The graft was oriented with the donor DM facing the recipient posterior stroma and attached onto the recipient posterior stroma with air. The anterior chamber was pressurized with air for 60 to 80 minutes, followed by an air-fluid exchange leaving a 50% air bubble.

Patients who experienced intraoperative and/or postoperative complications were excluded from the study. Complications were defined as any event that could potentially affect visual quality measurements: significant graft detachment or any detachment causing corneal edema (even if edema was not affecting the visual axis), the use of corneal sutures, paralytic mydriasis caused by ischaemia during the anterior chamber pressurization, the presence of significant amount of pigment on the intraocular lens, and delayed epithelial wound healing (more than 3 weeks). Other exclusion criteria were the presence of significant refractive errors prior to DMEK surgery (>5 diopters of spherical equivalent refraction or 3 diopters of astigmatism); axial length >26 mm or <22 mm; intraocular pressure ≥21 mmHg; media opacifications such as corneal fibrosis, cataract, or vitreous opacifications; concomitant ocular diseases, including history of glaucoma or retinal pathology; and systemic conditions that could affect the visual system. All controls included in the study were pseudophakic (uncomplicated surgery) and had no history nor evidence of ocular or neurologic disease of any nature; their best-corrected visual acuity (BCVA) was >20/30 based on the Snellen scale. Only one eye per subject was randomly selected in the control group and included. From a total of 20 consecutive patients planned for DMEK, 7 patients were excluded (2 due to significant detachment that produced corneal edema for longer than a month; 2 patients were excluded due to corneal epithelial ulcers with delayed healing; 1 due to iris ischaemia, 1 due to superficial corneal fibrosis, and 1 due to fibrotic maculopathy), and their data (preoperative and postoperative) were withdrawn from the final statistical analysis.

All patients underwent visual function and visual quality evaluation before surgery (from 1 week to a maximum of 2 months prior the intervention) and at one and six months after the DMEK procedure. Controls were evaluated in one visit, at least 6 months after cataract surgery. Visual function was assessed in all participants by evaluating BCVA using an Early Treatment Diabetic Retinopathy Study (ETDRS) chart and contrast sensitivity vision (CSV) using the Pelli-Robson and CSV-1000E tests. Structural corneal parameters were evaluated with the Pentacam® system (OCULUS, Wetzlar, Germany).

LogMAR visual acuity (VA) was assessed under monocular vision with best spectacle correction, in two different controlled lighting conditions: photopic (85 cd/m^2^) and mesopic (3 cd/m^2^). Contrast sensitivity provides more complete information about visual function than does visual acuity tests. CSV was evaluated in our patients using the Pelli-Robson chart and the CSV-1000E test. The Pelli-Robson chart comprises horizontal lines of capital letters organized into groups of three (triplets) with two triplets per line. The contrast decreases from one triplet to the next, even within each line. All patients were evaluated under monocular vision at a distance of 1 meter from the chart and under controlled photopic conditions (85 cd/m^2^). The score corresponding to the last triplet of letters seen by the patient was recorded. The CSV-1000E instrument is used worldwide for standardized CSV and glare testing. All patients were evaluated at a distance of 2.5 meters from the chart under monocular vision at 4 different spatial frequencies (3, 6, 12, and 18 cycles per degree (cpd)), under 3 different lighting conditions: photopic (85 cd/m^2^), mesopic (3 cd/m^2^), and mesopic with glare (3 cd/m^2^ + 90/100). The chart comprises four rows with 17 circular patches each. The patches present a grating that decreases in contrast moving from left to right across the row. The patient indicates whether the grating appears in the top patch or the bottom patch for each column. Each contrast value for each spatial frequency was transformed into a logarithmic scale according to standardized values.

Corneal quality parameters were evaluated using the Pentacam® system (OCULUS, Wetzlar, Germany). This device uses a rotational Scheimpflug camera that produces high-resolution three-dimensional images of the anterior pole of the eye. It provides different corneal maps (curvature, refraction, elevation, and pachymetric maps) and calculates numerical parameters of keratometry. Additionally, the software calculates corneal densitometry (backscattered light) in 3 different fixed corneal layers (anterior layer (anterior 120 mm), central layer, and posterior layer (posterior 60 mm)), as well as in fixed corneal concentric rings around the apex (central 0–2 mm, 2–6 mm, 6–10 mm, and 10–12 mm) [12].

For this study, central, anterior, and posterior corneal densitometry (0–2 mm zone and total); mean keratometry (Km); and the root mean square values (RMS) for total, low-order, and high-order aberrations (LOA and HOA, respectively) were calculated for anterior and posterior cornea and recorded. LOA include the second-order Zernike polynomials which represent the conventional aberrations defocus (myopia, hyperopia, and astigmatism). These aberrations represent 85% of total aberrations in the eye. HOA describe Zernike aberrations above second-order: third-order Zernike terms are coma and trefoil; fourth-order Zernike terms include spherical aberration. Higher-order aberrations make up about 15% of the overall number of aberrations in an eye and cannot be corrected by any means of present technology. Central corneal thickness and endothelial cell density at 6 months were also measured in our patients.

All data analyses were performed using SPSS software version 20.0 (SPSS Inc., Chicago, IL). To monitor the progression of corneal changes after DMEK, visual function and visual quality parameters were compared within the patients groups: preoperative data were compared with data obtained at one month after surgery, and the latter were compared with measurements obtained at 6 months after DMEK. To evaluate the differences between corneas which underwent DMEK surgery and healthy corneas, parameters obtained at 6 months after DMEK in patients were compared with measurements obtained in controls. Due to the nonparametric distribution of the data, comparisons between the different groups were calculated using the Mann–Whitney *U* test. A correlation analysis between visual function and topographic parameters was performed using Spearman's Rho test. A level of significance was considered at *p* < 0.05. To avoid a high false-positive rate, the Bonferroni correction for multiple tests was calculated, and the corrected *p* values were added to the previously calculated data.

## 3. Results

A total of 13 eyes in 13 different patients who underwent DMEK surgery and 14 eyes of 14 healthy controls were included in the study. Mean age in the patients group was 69.45 ± 7.51 years and in the control group was 72.62 ± 9.38 years (*p*=0.296). Mean axial length was 23.58 ± 1.74 mm in the DMEK group and 23.47 ± 1.25 mm in the control group (*p*=0.722). Anterior and posterior keratometric values were similar between both groups (*p*=0.841 and *p*=0.080, respectively). The indication for DMEK was Fuchs endothelial dystrophy (*n* = 8), bullous keratopathy (*n* = 2), or both (*n* = 3). At 6 months postoperative, mean central corneal thickness in patients was 507 ± 36 microns, and mean endothelial cell density was 912 ± 326 cells/mm^2^.

### 3.1. Improvement of Visual and Corneal Parameters after DMEK

Patients who underwent DMEK experienced a significant improvement in all visual function parameters at one month after surgery (Table 1). After the first postoperative month, all parameters continued to improve. However, only photopic BCVA (0.31 ± 0.19 at 1 month vs 0.13 ± 0.09 at 6 months, *p*=0.008), CSV at 6 cpd under mesopic conditions + glare (1.06 ± 0.58 at 1 month vs 1.46 ± 0.33 at 6 months, *p*=0.034), and CSV as measured with the Pelli-Robson chart (1.29 ± 0.18 at 1 month vs 1.48 ± 0.14 at 6 months, *p*=0.006) improved significantly at 6 months (Table 1).

A significant decrease of the 0–2 mm density both in the anterior (43.83 ± 10.50 preoperative vs 35.60 ± 12.16 at 1 month postoperative, *p*=0.045) and posterior cornea (27.70 ± 4.20 vs 22.07 ± 7.45, *p*=0.006) was observed at one month after DMEK. A significant improvement of the posterior Km was also observed at 1 month after surgery (−5.84 ± 0.23 preoperative vs −6.80 ± 0.67 at 1 month postoperative, *p*=0.005). Total corneal densities and posterior aberrations (LOA and total) also improved compared with preoperative levels without reaching significance. Posterior HOA did not change within the first month. A significant increase in the anterior corneal aberrations was observed at 1 month after DMEK (RMS LOA, 3.36 ± 1.23 preoperative vs 4.73 ± 1.67 at 1 month, *p*=0.026; RMS total aberrations, 3.60 ± 1.28 vs 5.04 ± 1.77, *p*=0.022) (Table 2).

Central corneal density (33.60 ± 13.87 at 1 month vs 24.32 ± 2.92 at 6 months, *p*=0.006), all anterior corneal densities (0–2, 35.60 ± 12.16 vs 25.21 ± 5.40, *p*=0.035; total, 38.40 ± 10.03 vs 29.25 ± 6.52 *p*=0.029), posterior 0–2 mm density (22.07 ± 7.45 vs 17.60 ± 2.83, *p*=0.010), and all posterior aberrations continued to decrease significantly at 6 months (RMS HOA, 0.69 ± 0.26 vs 0.47 ± 0.13, *p*=0.015; RMS LOA, 1.54 ± 0.56 vs 1.04 ± 0.29, *p*=0.020; RMS total 1.71 ± 0.57 vs 1.15 ± 0.29, *p*=0.015). Anterior corneal aberrations decreased at 6 months compared with 1 month after surgery. However, the differences did not reach significance levels (Table 2).

**Figure 1:**
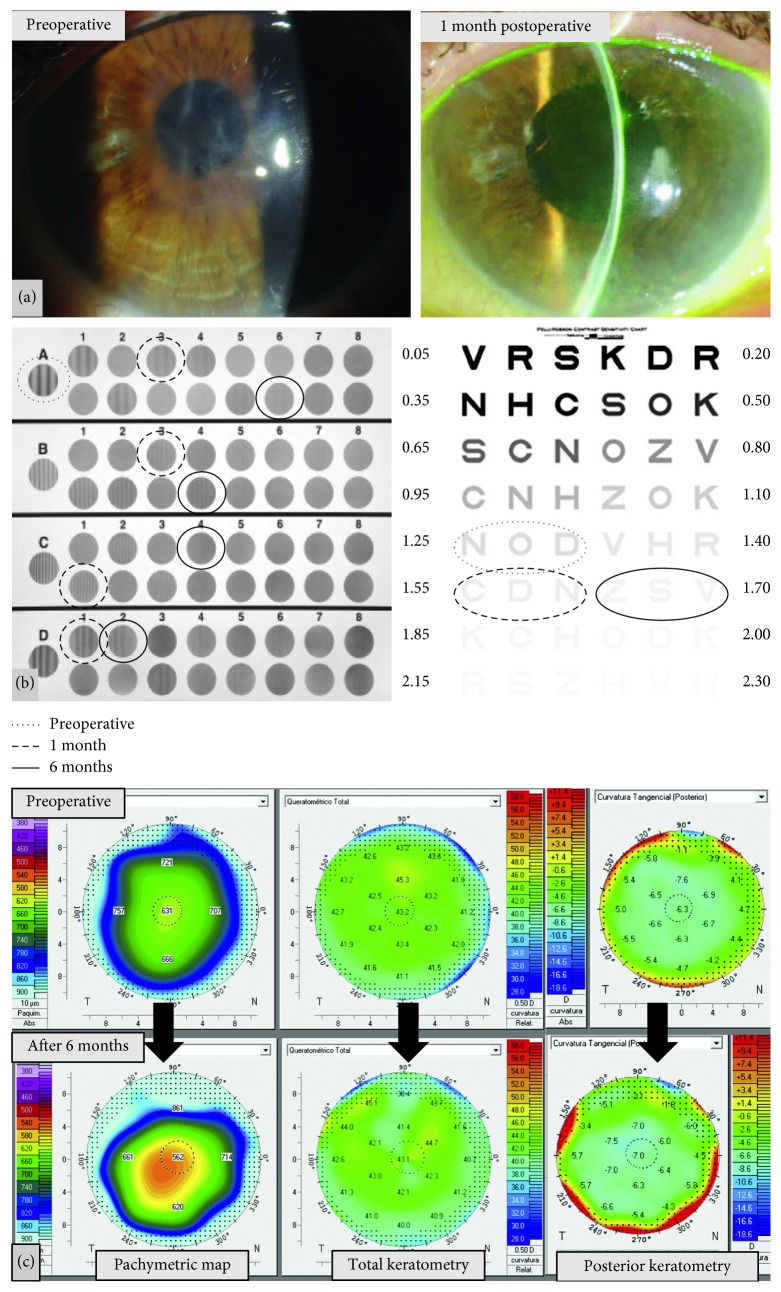
Representative case of a patient included in the study, who underwent DMEK. (a) Preoperative (left) and 1 month postoperative (right) slitlamp image of the right eye of a 62-year-old female patient who underwent DMEK. (b) Contrast sensitivity results: left, CSV 1000 test results marked with discontinuous-continuous circled lines (see legend in the figure); in frequencies B, C, and D, no preoperative circle was marked since the patient could not even identify the first image; right, Pelli-Robson results at preoperative and 1 month and 6 months postoperative. (c) Topographic changes preoperative and at 6 months postoperative: left, corneal thickness map; center, keratometric map of the frontal cornea; right, keratometric map of the posterior cornea.

A representative case of preoperative-postoperative changes after DMEK can be seen in Figure 1.

### 3.2. Comparison between DMEK Corneas and Healthy Corneas

Compared with healthy subjects, patients who underwent DMEK presented worse visual function at 6 months after DMEK, in all parameters except the CSV at 3 cpd (photopic, *p*=0.676; mesopic, *p*=0.064 and mesopic + glare, *p*=0.786) (Table 3).

No significant differences were observed between DMEK corneas at 6 months after surgery and healthy corneas in the central, anterior, and posterior corneal densitometry and in the anterior and posterior Km values (*p* > 0.05) (Table 4). Anterior (HOA, 1.50 ± 1.11 in patients vs 0.76 ± 0.21 in controls, *p*=0.021 and total 4.39 ± 2.23 vs 2.54 ± 0.74, *p*=0.014) and posterior (HOA, 0.47 ± 0.13 in patients vs 0.25 ± 0.10 in controls, *p* < 0.001; LOA, 1.04 ± 0.29 vs 0.67 ± 0.33, *p*=0.005 and total, 1.15 ± 0.29 vs 0.72 ± 0.34, *p*=0.002) aberrations remained higher in the group of DMEK patients compared with healthy controls (Table 4).

The correlation analysis did not reveal any significant association between visual function parameters and topographical changes in DMEK patients. An additional analysis was performed over a selected group of 8 patients who presented better visual results (BCVA≤0.1), and anterior and posterior HOAs were compared with controls. Patients with good visual outcomes 6 months after DMEK presented higher posterior HOAs compared with controls (0.62 ± 0.30 in DMEK vs 0.26 ± 0.11 in controls, *p*=0.001). However, though higher, no significant differences were observed in anterior HOAs between both groups (1.49 ± 0.70 in DMEK vs 0.78 ± 0.22 in controls, *p*=0.065).

### 3.3. Discussion

In the present study, we evaluated early visual rehabilitation and progressive corneal changes in 13 eyes which underwent DMEK surgery and compared them with a group of healthy subjects. Research on visual and corneal changes after DMEK typically focused on the outcomes at six months postoperative [9, 10, 13]. Despite published studies by the Melles team on early outcomes after DMEK, these results refer mainly to BCVA in photopic conditions [14, 15]. Measuring BCVA and contrast sensitivity in different lighting conditions may provide more accurate information about the visual system and the patient's possible performance in everyday situations (such as driving and reading) [16, 17]. Our patients' visual function (photopic and mesopic BCVA and CSV) improved dramatically after surgery, and most of the measured parameters stabilized at 1 month after the procedure. BCVA in photopic conditions additionally improved significantly at 6 months. Improvement in light scattering (both anterior and posterior) and posterior mean keratometry was observed in the first month. However, posterior HOA did not decrease until six months after surgery. Anterior HOAs did not change after DMEK in our patients.

Despite the observed changes and early improvement after DMEK, visual function at 6 months was worse than that in controls (except CSV in the 3 cpd frequency), and corneal HOA remained higher in patients than in healthy controls. These results support previous studies in which contrast sensitivity and posterior aberrations in eyes undergoing DMEK did not reach the same levels as controls [9, 13]. Garrido et al. demonstrated that CSV (as measured with the Pelli-Robson test) in pseudophakic patients undergoing DMEK remained worse than CSV in phakic healthy controls. Additionally, CSV 1000 test was used by Garrido et al. to assess CSV after DMEK and compare the results with other keratoplasty techniques [18]. DMEK demonstrated to preserve better CSV at 12 and 18 cpd compared with other procedures. However, these outcomes were never compared with a healthy population. Despite the numerous published articles on DMEK visual outcomes, we could not find any study performing a complete evaluation of visual function parameters (that is, measuring BCVA and CSV at different spatial frequencies and lighting situations) in DMEK patients compared with healthy controls. The present study provides not only a complete analysis of visual function changes after DMEK but also compares visual outcomes with a healthy population in similar circumstances.

Previous research by Van Dijk et al demonstrated that DMEK corneas presented a significant decrease in posterior HOA at six months after surgery, but as it was also observed in our patients, these aberrations remained higher compared with controls [9]. Rudolph et al. demonstrated that DMEK corneas presented higher HOA in the posterior 4 mm of the cornea compared to healthy eyes [10]. However, they failed to detect changes in anterior HOA and LOA, whereas van Dijk et al. found higher anterior HOA in their patients compared with controls [9]. Increased anterior and posterior HOAs were also observed recently in the 6 mm central cornea, in DMEK patients compared with controls [19]. Our patients did not experience any significant changes in anterior HAO; however, anterior LOA increased significantly in the first month, contrary to that observed by van Dijk et al. and Rudolph et al. [9, 10]. HOAs have been correlated to visual acuity after EK and PKP due to the degradation by HOAs of the small-angle domain of the retinal point-spread function [7, 19]. Posterior corneal HOAs increased after EK compared with healthy controls [19–23], and it has been suggested that the posterior corneal surface is the source of increased whole-eye HOAs after Descemet stripping endothelial keratoplasty (DSEK) compared with normal eyes [21, 24]. Posterior HOAs have been linked to BCVA after EK [19]; however, several studies have failed to find an association between posterior corneal HOAs and postoperative BCVA [9, 23, 25, 26], leading some authors to suggest that changes in the posterior cornea should not affect visual acuity [21, 27]. Anterior HOAs have been found to be higher after DSAEK than in normal corneas [28], and a significant correlation has been demonstrated between anterior corneal HOAs and postoperative BCVA [10, 22, 23]. Since EK itself causes minimal disruption of the anterior corneal surface, it is reasonable to suggest that other sources (that is, other than the surgical technique) of increased HOAs must exist in these patients, such as factors related to the underlying disease. In our patients, anterior HOAs after DMEK remained higher than in controls, and a significant increase in anterior LOAs was observed in the first month, whereas visual function and corneal densities improved. These observed changes might be due to preexistent chronic stromal edema, degeneration of keratocytes, and collagen reorganization after DMEK. Additionally, when our patients with better visual outcomes were analysed separately, no differences in anterior HOAs were observed compared with controls. This might also suggest that anterior HOAs play a more important role concerning BCVA results in these patients than do posterior HOAs. These results should still be analysed with caution due to the small sample size and the limitation to the statistical calculations.

Though corneal aberrations have been widely studied after EK, literature on light scattering after DMEK is scarce. We could only find one published study in which corneal density after DMEK was analysed and compared with healthy corneas [9]. In their study, Van Dijk et al. found a strong significant correlation between anterior corneal haze and postoperative BCVA.

It has been argued that light scatter alone cannot affect high-contrast visual acuity [6, 29]. However, increased light scatter may reduce visual quality after EK [29–31], and this is more evident in everyday low-contrast situations [21]. The anterior recipient cornea has been proved to be the main source of haze after other EK techniques such as DSEK and deep lamellar endothelial keratoplasty [6, 29]. Changes associated with this stromal haze seem to be independent of preoperative edema or fibrosis [32]. In our patients, corneal 0–2 mm anterior and posterior densities decreased significantly in the first month after DMEK. These early changes in light scattering had not been documented before since most DMEK studies evaluate their outcomes at 6 months after the procedure. All densities in our patients improved at 6 months after surgery reaching similar light scattering levels to healthy corneas. However, visual function outcomes remained worse than controls, suggesting that other factors such as anterior and posterior HOAs may be limiting postoperative visual quality outcomes in our patients. Our results differ from previous observations on light scattering after DMEK, where corneal densities at 6 months postoperative remained higher than those in healthy corneas. More studies evaluating changes in corneal density after DMEK are needed to corroborate our findings.

The most important limitation to our study is the small sample size, which may be limiting the statistical findings. We believe that correlations between visual results and corneal parameters were not observed in our study due to the small sample size, and further studies analysing visual function with a larger number of patients are needed to establish a correlation between BCVA and CSV and topographic changes in these patients. Given the large samples included in other studies (especially those from the Melles group), our study should be interpreted with caution when compared with other similar research studies, and factors responsible for visual function outcomes in our patients cannot be taken further from speculation.

## 4. Conclusions

Patients undergoing DMEK present visual function improvement and a decrease in anterior and posterior corneal density at 1 month after the procedure. A further decrease in corneal posterior HOAs was observed at 6 months. Despite this remarkable improvement, visual function outcomes remained worse compared with healthy corneas. Corneal parameters such as mean keratometry and corneal density were comparable to controls; however, HOAs remained higher in DMEK patients at 6 months after surgery. Similar studies with a larger simple size are needed in order to establish a possible correlation between visual function outcomes and corneal parameters after DMEK.

## Figures and Tables

**Table 1:** Visual function parameters in patients undergoing DMEK as measured preoperative and at 1 month and at 6 months postoperative.

Functional parameter	Preoperative	1 month postoperative	*P* (preoperative vs 1 month postoperative)	6 month postoperative	*P* (1 month vs 6 months postoperative)
*Visual Acuity*					
VA ETDRS photopic	0.59 (0.33)	0.31 (0.19)	**0.013**	0.13 (0.09)	**0.008**
VA ETDRS mesopic	0.75 (0.25)	0.50 (0.22)	**0.012**	0.36 (0.15)	0.130

*Contrast sensitivity*					
CSV 3cpd	0.46 (0.52)	1.06 (0.61)	**0.005**	1.49 (0.45)	0.077
CSV 6cpd	0.23 (0.52)	1.17 (0.65)	**<0.001** ^*∗*^	1.33 (0.47)	0.363
CSV12cpd	0.21 (0.41)	0.58 (0.50)	**0.031**	0.80 (0.46)	0.217
CSV18cpd	0.08 (0.18)	0.29 (0.31)	**0.015**	0.45 (0.46)	0.264
CSV-M 3cpd	0.44 (0.48)	1.20 (0.48)	**0.001** ^*∗*^	1.52 (0.28)	0.056
CSV-M 6cpd	0.19 (0.44)	1.19 (0.55)	**<0.001** ^*∗*^	1.26 (0.66)	0.401
CSV-M 12cpd	0.13 (0.31)	0.71 (0.67)	**0.004**	0.76 (0.56)	0.741
CSV-M 18cpd	0.07 (0.18)	0.23 (0.30)	**0.022**	0.34 (0.28)	0.350
CSV-MG 3cpd	0.21 (0.45)	1.06 (0.58)	**0.001** ^*∗*^	1.46 (0.33)	**0.034**
CSV-MG 6cpd	0.12 (0.35)	0.83 (0.57)	**<0.001** ^*∗*^	0.84 (0.64)	0.867
CSV-MG 12cpd	0.04 (0.15)	0.43 (0.39)	**0.001** ^*∗*^	0.59 (0.56)	0.434
CSV-MG 18cpd	0.05 (0.35)	0.27 (0.33)	**0.002** ^*∗*^	0.34 (0.28)	0.755
Pelli-Robson	1.00 (0.29)	1.29 (0.18)	**0.005**	1.48 (0.14)	**0.006**

*P* values correspond to comparisons preoperative versus 1 month and 1 month versus 6 months. Bold letters indicate *p* < 0.05. Asterisks mark Bonferroni values less than 0.003. DMEK, Descemet membrane endothelial keratoplasty; VA, visual acuity; ETDRS, Early Treatment Diabetic Retinopathy Study; CSV, contrast sensitivity vision; cpd, cycles per degree.

**Table 2:** Visual quality parameters as obtained with Oculus Pentacam of corneas undergoing Descemet membrane endothelial keratoplasty (DMEK) measured preoperative and at 1 month and at 6 months postoperative.

Quality parameter	Preoperative	1 month postoperative	*P* (preoperative vs 1 month postoperative)	6 months postoperative	*P* (1 month vs 6 months postoperative)
*Anterior cornea*					
Central density	39.81 (10.99)	33.60 (13.87)	0.069	24.32 (2.92)	**0.006** ^*∗*^
0–2 mm density	43.83 (10.50)	35.60 (12.16)	**0.045**	25.21 (5.40)	**0.035**
Total density	41.06 (10.43)	38.40 (10.03)	0.489	29.25 (6.52)	**0.029**
Km	43.83 (2.03)	43.13 (1.86)	0.281	42.85 (1.50)	0.607
RMS HOA (*µ*m)	1.19 (0.52)	1.66 (0.79)	0.249	1.50 (1.11)	0.317
RMS LOA (*µ*m)	3.36 (1.23)	4.73 (1.67)	**0.026**	3.86 (2.24)	0.427
RMS total (*µ*m)	3.60 (1.28)	5.04 (1.77)	**0.022**	4.39 (2.23)	0.522

*Posterior cornea*					
0–2 mm density	27.70 (4.20)	22.07 (7.45)	**0.006** ^*∗*^	17.60 (2.83)	**0.010**
Total density	29.06 (4.56)	26.26 (5.44)	0.214	23.38 (3.52)	0.128
Km	−5.84 (0.23)	−6.80 (0.67)	**0.005** ^*∗*^	−6.52 (0.39)	0.217
RMS HOA (*µ*m)	0.69 (0.34)	0.69 (0.26)	0.828	0.47 (0.13)	**0.015**
RMS LOA (*µ*m)	1.71 (1.08)	1.54 (0.56)	0.870	1.04 (0.29)	**0.020**
RMS total (*µ*m)	1.86 (1.11)	1.71 (0.57)	0.703	1.15 (0.29)	**0.015**

Bold letters indicate *p* < 0.05. Asterisk marks Bonferroni values <0.007. DMEK, Descemet membrane endothelial keratoplasty; Km, mean keratometry; RMS, root mean square; HOA, high-order aberrations; LOA, low-order aberrations; *µ*m, microns.

**Table 3:** Visual function parameters in patients undergoing Descemet membrane endothelial keratoplasty (DMEK) at 6 months postoperative compared with healthy corneas.

Functional parameter	DMEK	Controls	*p*
*Visual Acuity*			
VA ETDRS photopic	0.13	−0.15	**0.002** ^*∗*^
VA ETDRS mesopic	0.36	0.23	**0.044**

*Contrast sensitivity*			
CSV 3cpd	1.49	1.62	0.676
CSV 6cpd	1.33	1.79	**<0.001** ^*∗*^
CSV 12cpd	0.80	1.39	**0.006**
CSV 18cpd	0.45	0.95	**0.006**
CSV-M 3cpd	1.52	1.72	0.064
CSV-M 6cpd	1.26	1.81	**0.005**
CSV-M 12cpd	0.76	1.46	**0.001** ^*∗*^
CSV-M 18cpd	0.34	0.94	**<0.001** ^*∗*^
CSV-MG 3cpd	1.46	1.61	0.786
CSV-MG 6cpd	0.84	1.63	**0.001** ^*∗*^
CSV-MG 12cpd	0.59	1.33	**0.001** ^*∗*^
CSV-MG 18cpd	0.39	1.10	**0.002** ^*∗*^
Pelli-Robson	1.48	1.71	**0.002** ^*∗*^

Bold letters indicate *p* < 0.05. Asterisks mark Bonferroni values less than 0.003. DMEK, Descemet membrane endothelial keratoplasty; VA, visual acuity; ETDRS, Early Treatment Diabetic Retinopathy Study; CSV, contrast sensitivity vision; cpd, cycles per degree.

**Table 4:** Visual quality parameters as obtained with Oculus Pentacam of corneas undergoing Descemet membrane endothelial keratoplasty (DMEK) at six months after surgery compared with healthy controls.

Quality parameter	DMEK	Controls	*p*
*Anterior cornea*			
Central density	24.32 (2.92)	23.84 (9.87)	0.099
0–2 mm density	25.21 (5.40)	29.83 (9.30)	0.275
Total density	29.25 (6.52)	36.35 (9.54)	0.052
Km	42.85 (1.50)	44.15 (1.77)	0.058
RMS HOA (*µ*m)	1.50 (1.11)	0.76 (0.21)	**0.021**
RMS LOA (*µ*m)	3.86 (2.24)	2.42 (0.73)	0.069
RMS total (*µ*m)	4.39 (2.23)	2.54 (0.74)	**0.014**

*Posterior cornea*			
0–2 mm density	17.60 (2.83)	18.46 (6.51)	0.734
Total density	23.38 (3.52)	26.72 (6.33)	0.234
Km	−6.52 (0.39)	−6.36 (0.24)	0.292
RMS HOA (*µ*m)	0.47 (0.13)	0.25 (0.10)	**<0.001**
RMS LOA (*µ*m)	1.04 (0.29)	0.67 (0.33)	**0.005**
RMS total (*µ*m)	1.15 (0.29)	0.72 (0.34)	**0.002**

Central density has been included in the anterior corneal measurements group. Bold letters indicate *p* < 0.05. DMEK, Descemet membrane endothelial keratoplasty; Km, mean keratometry; RMS, root mean square; HOA, high-order aberrations; LOA, low-order aberrations; *µ*m, microns.

## Data Availability

The data used to support the findings of this study are available from the corresponding author upon request.
